# Comparison of the Pathogenicity of Two Different Branches of Senecavirus a Strain in China

**DOI:** 10.3390/pathogens9010039

**Published:** 2020-01-02

**Authors:** Huawei Zhang, Pin Chen, Genxi Hao, Wenqiang Liu, Huanchun Chen, Ping Qian, Xiangmin Li

**Affiliations:** 1 State Key Laboratory of Agricultural Microbiology, Huazhong Agricultural University, Wuhan 430070, Chinahaogengxi@webmail.hzau.edu.cn (G.H.); liuwenqiang1993@webmail.hzau.edu.cn (W.L.); chenhch@mail.hzau.edu.cn (H.C.); 2 Laboratory of Animal Virology, College of Veterinary Medicine, Huazhong Agricultural University, Wuhan 430070, China; chenpin@mail.hzau.edu.cn; 3 Key Laboratory of Development of Veterinary Diagnostic Products, Ministry of Agriculture, Wuhan 430070, China; 4 Key Laboratory of Preventive Veterinary Medicine in Hubei Province, The Cooperative Innovation Center for Sustainable Pig Production, Wuhan 430070, China

**Keywords:** Senecavirus A (SVA), emerging disease, PIVD, pathogenicity, China

## Abstract

Senecavirus A (SVA), an emerging infectious disease, is associated with the porcine idiopathic vesicular disease. Here, the pathogenesis of different strains of SVA was investigated in growing-finishing pigs. We aimed to evaluate the replication characteristics, virus particle morphology, clinical signs, and vesicular lesions in comparison with two different strains of SVA. The animals were infected with SVA HB-CH-2016 or CH/AH-02/2017 by intranasal routes (3 mL, 10^9^TCID_50_/mL) and monitored daily for 14 days post-inoculation (dpi) for clinical signs and vesicular lesions. Viremia or viral shedding was detected in the blood, fecal swab, and nasal swab samples. Results showed no distinct differences in plaque size, replication ability, and characteristic virions between SVA HB-CH-2016 and CH/AH-02/2017 strains. Animal experimental results showed that both SVA CH/AH-02/2017 and SVA HB-CH-2016 could infect pigs. However, an obvious difference in the pathogenicity and dynamics of infection was observed between SVA HB-CH-2016 and CH/AH-02/2017 strains. The pathogenesis of SVA CH/AH-02/2017 was similar to that of published results of USA strains, whereas the SVA HB-CH-2016 strain had low pathogenicity to pigs. Clinical signs and vesicular lesions were observed in SVA CH/AH-02/2017-infected pigs. Additionally, the different branches of SVA should be capable of inducing broad cross-reactive neutralizing antibodies, which play an important role in clearing the SVA virus. This study of animal models for SVA infection will be beneficial to develop vaccines and antivirals.

## 1. Introduction

Senecavirus A (SVA), also called the Seneca valley virus, is a member of the genus Senecavirus from the family Picornaviridae [1]. The virus is a positive, single-strand RNA virus. The genome of SVA is approximately 7.2 kb in length, which contains a single open reading frame (ORF). The ORF encoding a polyprotein of 2181 amino acids, consists of 12 proteins (Lpro, VP1, VP2, VP3, VP4, 2A, 2B, 2C, 3A, 3B, 3C, and 3D) [1,2,3]. Structural biology analysis revealed that the B-cell epitopes of SVA mainly exist in VP2 and VP3 proteins (in our laboratory). Therefore, the VP2 and VP3 proteins are important targets for designing gene engineering vaccines and diagnostic agents.

As an emerging infectious disease, SVA is associated with the porcine idiopathic vesicular disease (PIVD) [4]. SVA causes acute lameness and vesicular lesions on the coronary bands, hooves, or snouts in pigs [4,5,6]. SVA infection mainly leads to an outbreak of the disease in sows, which affects the production of pigs [7,8,9]. Currently, the main strategies used to control SVA depend on biosecurity measures. Chinese scholars have developed inactivated SVA vaccines to protect pigs against SVA infection [10].

**Figure 1:**
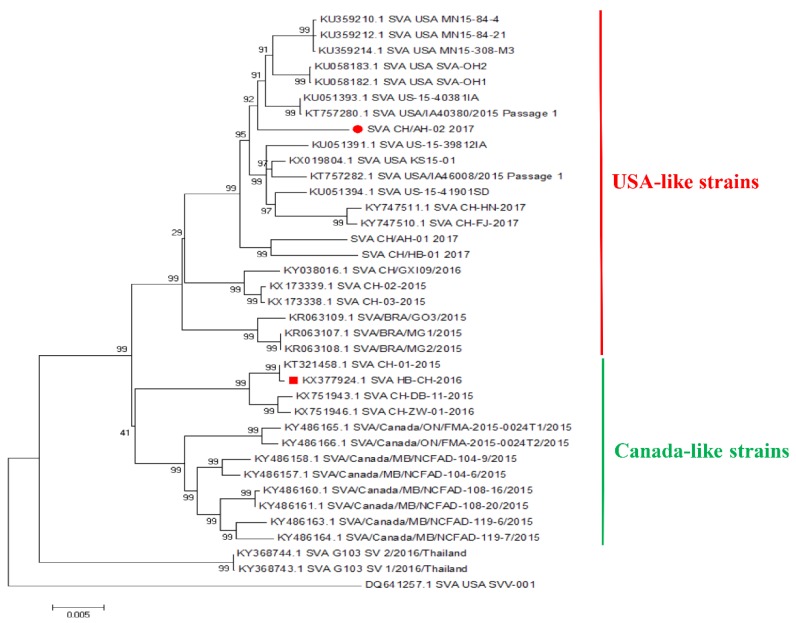
Phylogenetic analysis based on the complete genomes of the isolated virus. Phylogenetic Table 7. 0.18 software with the method of the neighbor-joining (1000 bootstrap replicates). The isolated virus was marked filled red circle.

SVV-001 was the first SVA isolated from PER.C6 cells contaminated in 2002 [1]. SVV-001 is non-virulent to humans, and it was developed as an oncolytic agent to treat neuroendocrine tumors in humans [11,12,13,14]. SVV-001 can infect pigs but is not pathogenic, which indicates that it has no virulence to pigs [15]. In recent years, outbreaks of SVA infection have been reported worldwide, and many different virulent strains were isolated from clinical cases. In 2015, the first isolated virus of SVA infection was identified in China [16]. In a previous study, our research group reported an SVA strain isolated in the Hubei Province of China in 2016 [17]. To date, outbreaks of SVA infection are reported in many provinces in China. This situation means that SVA is widely found in pig farms in China [16,17,18,19]. Phylogenetic analysis indicated that SVA strains in pigs in China have five different types [20]. Hence, we further analyzed Chinese SVA strains belonging to USA-like SVA strains or Canada-like strains (Figure 1). Since 2017, isolated Chinese SVA strains were found to belong to USA-like strains, suggesting that USA-like SVA strains are predominant in China [6]. 

In our laboratory, the identified SVA HB-CH-2016 was found to belong to Canada-like strains in 2016. SVA CH/AH-02/2017 was also isolated and identified to belong to USA-like strains in 2017. In the present study, we compared the pathogenicity of the SVA HB-CH-2016 and SVA CH/AH-02/2017 strains via the intranasal route in growing-finishing pigs. We aimed to analyze the biological characteristics and evaluate viral infections of these two strains from different branches of SVA strains and provide a good foundation for the development of a vaccine.

## 2. Materials and Methods 

### 2.1. Cells and Viruses

Porcine kidney cells (SK-6, CCTCC) were grown in Dulbecco’s modified essential medium (DMEM, Invitrogen, Waltham, MA, USA) containing 10% fetal bovine serum (FBS, Gibco, Waltham, MA, USA) at 37 °C in a humidified 5% CO_2_ incubator. The SVA HB-CH-2016 (GenBank Accession No. KX377924.1) and SVA CH/AH-02/2017 strains (GenBank Accession No. MF460449) were isolated in China on Hamster Kidney cells (BHK-21, ATCC). In this research, both SVA HB-CH-2016 and CH/AH-02/2017 strains were amplified and titrated in SK-6 cells.

### 2.2. Genomic Sequence Analysis and Phylogenetic Analysis

Pairwise nucleotide and amino acid sequence identities between SVA HB-CH-2016 and SVA CH/AH-02/2017 were analyzed using the Clustal W method of the MEGA 7.0 software (https://www.megasoftware.net/). The phylogenetic trees of complete genomes were constructed through the neighbor-joining method, and the maximum composite likelihood model was established using 1000 replicates with the bootstrap values.

### 2.3. One-Step Growth Curve

The confluent monolayers of SK-6 cells in a six-well cell culture plate were washed twice with PBS and then inoculated with the SVA HB-CH-2016 or SVA CH/AH-02/2017 strain at a multiplicity of infection (MOI) of 0.1. The cells were incubated at 37 °C for 1 h, and the inoculum was discarded and washed twice with PBS. Fresh DMEM, containing 2% FBS was then added. The cells were cultured in an incubator at 37 °C. The cell culture was harvested at 0, 12, 24, and 36 h. After three freeze-thaw cycles, the cultures were harvested by centrifugation and titrated by plaque assay or 50% tissue culture infective dose (TCID_50_) assay in SK-6 cells. 

### 2.4. Electron Microscopy

SK-6 cells were infected with the SVA HB-CH-2016 or SVA CH/AH-02/2017 strain at an MOI of 0.1. After incubation for 36 h, the cultures were harvested by centrifugation. Subsequently, the supernatant was centrifuged at 160,000× *g* for 4 h with a Beckman SW32Ti rotor by using a 20% (w/v) sucrose cushion. The pellets were resuspended in PBS and then centrifuged with 20%, 35%, 50%, and 65% sucrose discontinuous gradient with a Beckman SW41Ti rotor. Viral bands were collected at 50% sucrose concentration and resuspended in PBS. The pellets were centrifuged with PBS at 160,000× *g* for 4 h with a Beckman SW41Ti rotor. Purified virions were confirmed by 12% sodium dodecyl sulfate-polyacrylamide gel electrophoresis (SDS-PAGE). Purified virions (approximately 4 μL) were adsorbed onto a carbon-coated copper grid for 4 min at room temperature. After washing three times with 2% uranyl acetate, the excess liquid was removed with filter paper. Then, samples were observed by using an H-7000FA electron microscope (HITACHI, Tokyo, Japan).

### 2.5. Experimental Infection of Pigs

The animal experiment was approved by the Research Ethics Committee of College of Veterinary Medicine, Huazhong Agricultural University, Hubei, China. Ten large white growing-finishing pigs of 90–100 kg were purchased from the experimental farm of Huazhong Agricultural University and randomly divided into two groups, namely, the SVA HB-CH-2016 and SVA CH/AH-02/2017 groups. All large white growing-finishing pigs (castrated hog) were confirmed to be seronegative for SVA by the neutralization assay. The SVA HB-CH-2016 and SVA CH/AH-02/2017 groups of large white growing-finishing pigs were placed in separate rooms to avoid cross-contamination. Pigs in the SVA HB-CH-2016 group were challenged with 3 mL of SVA HB-CH-2016 (10^9^TCID_50_/mL) by intranasal (1.5 mL to each nostril) routes. Pigs in the SVA CH/AH-02/2017 group were challenged with 3 mL of CH/AH-02/2017 (10^9^TCID_50_/mL) by intranasal (1.5 mL to each nostril) routes. Following the challenge, the rectal temperature and clinical signs (lethargy, lameness, and vesicular lesions) were monitored daily throughout the experiment. The clinical scores were used to evaluate vesicular lesions following previously established standards [10]. Clinical scores were calculated as follows: no symptoms, 0 points; each foot appeared lesions, 1 point; and vesicular lesions appeared in or around the mouth, 1 point. Hence, the maximum total score per pig was five. The blood, nasal swab, and fecal swab samples were collected at 0, 2, 4, 6, 8,10,12, and 14 days post-inoculation (dpi). 

### 2.6. Quantitative Real-Time PCR

RNA of the blood, fecal swab, and nasal swab samples was isolated using the TRIzol reagent (Invitrogen, USA) according to the manufacturer’s instructions. SVA quantitative reverse transcription-polymerase chain reaction (qRT-PCR) was performed as previously described [10]. SVA 3D primers (SVA 3D-F: 5′- AGAATTTGGAAGCCATGCTCT-3′; SVA 3D-R: 5′-GAGCCAACATAGATACAGATTGC-3′) were synthesized, and the TaqMan probe was 5′-FAM-TTCAAACCAGGAACACTACTCGAG-TAMRA-3′. qRT-PCR was performed using the THUNDERBIRD Probe qPCR Mix (TOYOBO Biotechnology Co. Ltd., Shanghai). Viral genome copy numbers were determined using the standard curve, and the results were expressed as log_10_RNA copies/mL.

### 2.7. Cross-Neutralization Test

The neutralization assay was performed as follows. The serum samples of SVA HB-CH-2016 or SVA CH/AH-02/2017 from inoculated animals were heat-inactivated at 56 °C for 30 min. The serum was diluted in a twofold serial dilution and incubated with 50 μL of 200 TCID_50_ SVA HB-CH-2016 or SVA CH/AH-02/2017 for 1 h at 37 °C. Subsequently, 100 μL of 10^6^ cells/mL SK-6 cell in DMEM containing 2% FBS was added to each well. The neutralizing antibody titer was expressed as the reciprocal of the highest dilution at which more than 50% of virus growth was inhibited.

### 2.8. Histopathological Examination and Immunohistochemistry (IHC)

At 14 dpi, pigs from each group were euthanized. During necropsy, the collected organs were subjected to pathological and IHC examination. Collected samples were fixed in 10% PBS buffered formalin for 24–36 h, dehydrated by different ethanol concentrations, and fixed in paraffin and sectioned. HE staining and Immunohistochemical method were performed in thin sections with 3–6 μm thickness. IHC slices were incubated with SVA VP1-specific rabbit polyclonal (prepared in our laboratory, 1:200 dilution in PBS) and then incubated with peroxidase-conjugated goat anti-rabbit Immunoglobulin G (Sigma, diluted 1:500 dilution in PBS).

### 2.9. Statistical Analysis

Statistical analyses were performed using one-way ANOVA in the GraphPad Prism software (GraphPad Software Inc., La Jolla, CA, USA). *p* < 0.05 was considered statistically significant.

## 3. Results

### 3.1. Genomic Sequence Comparison between SVA HB-CH-2016 and SVA CH/AH-02/2017 Strains

The analysis of the complete genomic sequence showed that SVA HB-CH-2016 shared 96% sequence identity with SVA CH/AH-02/2017. Sequence analysis demonstrated that the 29 amino acids different between the two strains (Table 1). Phylogenetic analysis indicated that SVA HB-CH-2016 belonged to Canada-like strains, and SVA CH/AH-02/2017 belonged to USA-like SVA strains (Figure 1). Since 2017, USA-like SVA strains have become predominant in China.

### 3.2. Replication Kinetics of the SVA HB-CH-2016 and SVA CH/AH-02/2017 Strains

**Figure 2:**
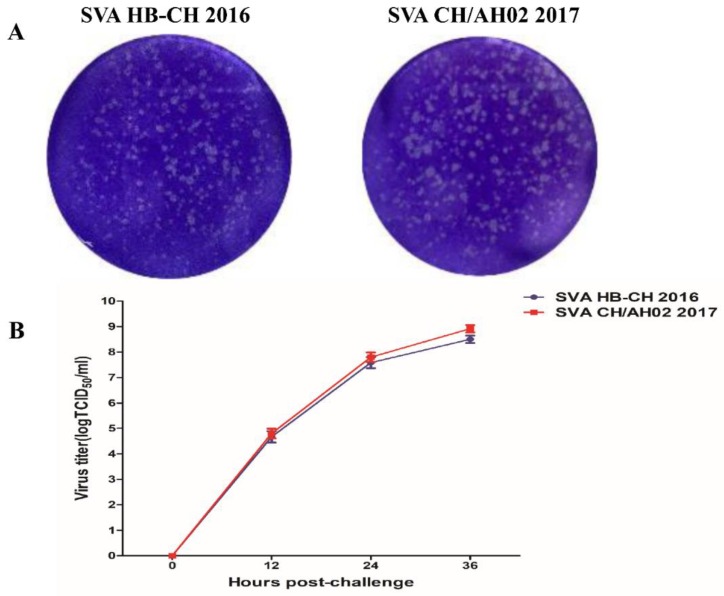
Replication characteristics of the SVA HB-CH-2016 and SVA CH/AH-02/2017 strains were compared in porcine cells. (**A**) Plaque morphology of SVA HB-CH-2016 and SVA CH/AH-02/2017. (**B**) One-step growth curves of SVA HB-CH-2016 and SVA CH/AH-02/2017. All data were expressed as mean ± SEM.

To analyze the replication characteristics of the SVA HB-CH-2016 and SVA CH/AH-02/2017 strains, we performed plaque formation tests and one-step growth experiments. As shown in Figure 2A, no significant difference was observed in the plaque size of SVA, which was approximately 0.1–0.2 mm in diameter. SVA HB-CH-2016 displayed a similar replication level to SVA CH/AH-02/2017. At 36 h, the two SVA viral titers exceeded 10^8.0^ TCID_50_/mL (Figure 2B).

### 3.3. Electron Microscopy

**Figure 3:**
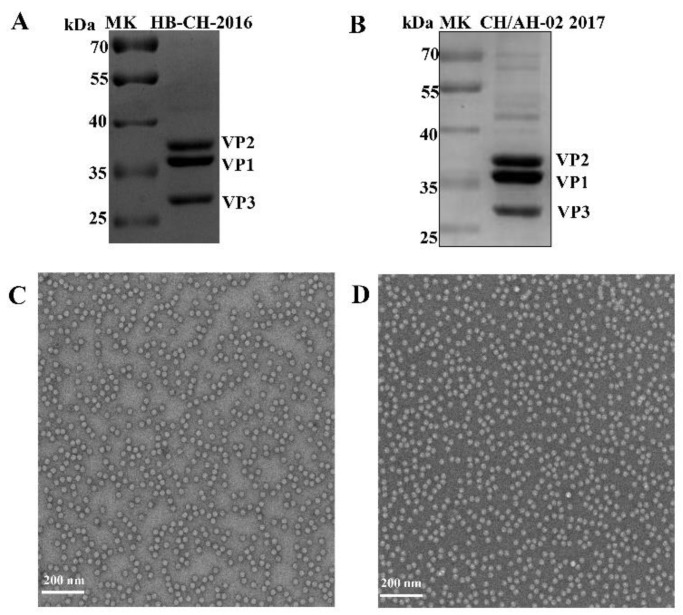
SVA morphology was identified by electron microscopy (HITACHI, Tokyo, Japan). Purified (**A**) SVA HB-CH-2016 and (**B**) SVA CH/AH-02/2017 were analyzed by 12% sodium dodecyl sulfate-polyacrylamide gel electrophoresis (SDS-PAGE). Electron microscopy of negatively stained purified virus (**C**) SVA HB-CH-2016 and (**D**) SVA CH/AH-02/2017. Scale bar indicates 200 nm.

The SVA HB-CH-2016 and SVA CH/AH-02/2017 strains were cultured in SK6 cells. Approximately 500 mL of viral supernatant was harvested by centrifugation. The virions of SVA HB-CH-2016 or SVA CH/AH-02/2017 were purified successfully by high-speed centrifugation in sucrose gradient. SDS-PAGE was applied to analyze the purification of SVA. The SVA HB-CH-2016 and SVA CH/AH-02/2017 strains of capsid proteins (VP2, VP1, and VP3 proteins) were observed (Figure 3A,B). The purification of SVA was examined by electron microscopy (HITACHI, Tokyo, Japan). Results of electron microscopy showed that the virions of SVA HB-CH-2016 (Figure 3C) and SVA CH/AH-02/2017 (Figure 3D) were round, and the diameter of virions was approximately 30 nm. 

### 3.4. Pathogenicity of the SVA HB-CH-2016 and SVA CH/AH-02/2017 Strains

**Figure 4:**
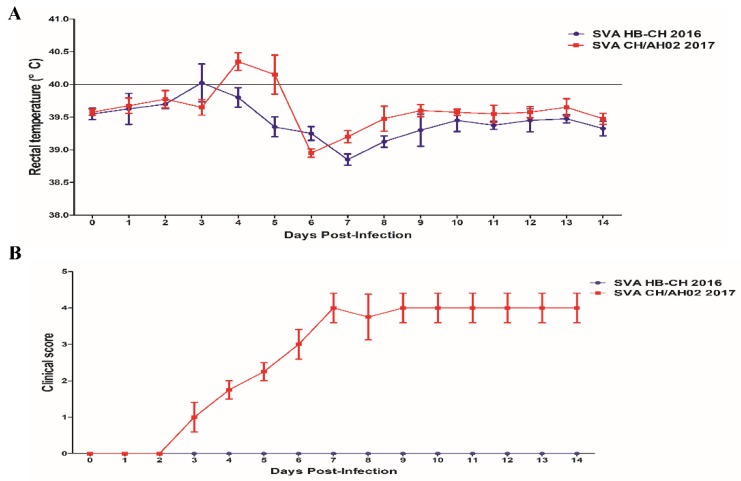
Rectal temperatures (**A**) and clinical scores (**B**) of the pigs infected with SVA HB-CH-2016 and SVA CH/AH-02/2017. All data were expressed as mean ± SEM.

**Figure 5:**
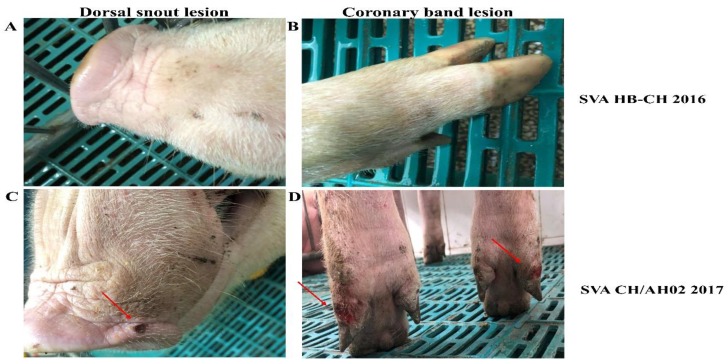
Vesicular lesion observed on pigs infected with SVA CH/AH-02/2017. All pigs infected with SVA HB-CH-2016 remained healthy, and no signs of disease were found throughout the experiment. (**A**) The upper lip of an SVA HB-CH-2016-infected pig; (**B**) Feet of an SVA HB-CH-2016-infected pig. Vesicular lesion observed on the upper lip (**C**) and feet (**D**) of an SVA CH/AH-02/2017-infected pig.

To compare the pathogenicity of SVA HB-CH-2016 and SVA CH/AH-02/2017, we divided 10 large white growing-finishing pigs of 90–100 kg into two groups: the SVA HB-CH-2016 and SVA CH/AH-02/2017 groups. In each group, the pigs were infected with the same dose of SVA. The rectal temperature and clinical signs of infection were periodically monitored daily throughout the experiment. No clinical signs were observed in the SVA HB-CH-2016-infected pigs, and the average rectal temperature of pigs did not exceed 40.0 °C during the experiments. After the experiment, all SVA HB-CH-2016-infected pigs remained healthy, and no signs of disease were found. The temperature of all pigs in the SVA CH/AH-02/2017 group slightly elevated to 40 °C at 4 and 5 dpi (Figure 4A). The clinical signs (lameness and vesicular lesions) were observed at 3 dpi, which continued until the end of the experiment. On the basis of the clinical scoring method for SVA, as described previously [10], each pig was recorded daily after the experimental infection. As shown in Figure 4B, the clinical score of the SVA CH/AH-02/2017 group was calculated as 4. At 5 dpi, fluid-filled vesicles on the snout burst, and erosion on the upper lip was observed in pigs infected with SVA CH/AH-02/2017 (Figure 5). Thereafter, no new vesicular lesions were detected in any of the lips. At the same time, gross ulcerative lesions were detected on the feet. Throughout the experiment, the SVA HB-CH-2016 group did not present clinical signs and vesicular lesions. At 8 dpi, one pig from each group was euthanized. No apparent gross lesions at necropsy were found in either group, except for vesicular lesions in the SVA CH/AH-02/2017 group. These results indicated that the SVA CH/AH-02/2017 strain displayed higher pathogenicity to pigs than the SVA HB-CH-2016 strain.

### 3.5. Histopathological Lesions

**Figure 6:**
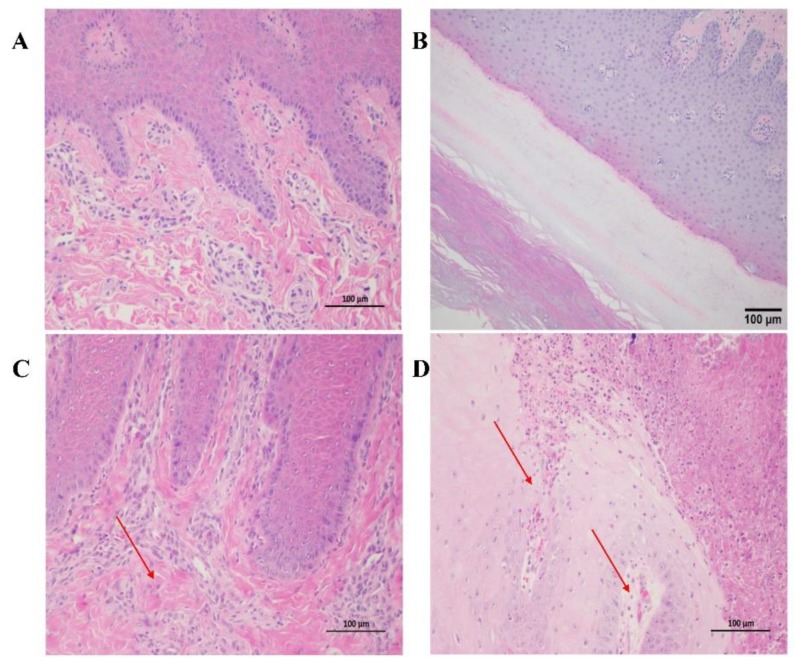
Histopathological examination of the snout and coronary band of pigs infected with the SVA HB-CH-2016 and SVA CH/AH-02/2017 strains. No significant histopathological lesions were observed in the snout (**A**) and coronary band (**B**) of pigs infected with SVA HB-CH-2016. Histological examination of the nasolabial skin (**C**) and coronary band epidermis (**D**) showed moderate or severe necrosis, and the epithelial cells of necrotic foci were swollen in pigs infected with SVA CH/AH-02/2017. Original magnification, 200×.

Pigs from each group underwent humane killing at 14 dpi, and pathological changes were described. No evidence of lesions at necropsy was found in the visceral (internal) organs. All the pigs in the SVA CH/AH-02/2017 group showed vesicular lesions on the feet or snout. Histopathological analyses were performed in the region of the lesion of the feet or snout. No histopathological lesions were observed in the lips and hooves of pigs infected with SVA HB-CH-2016 (Figure 6A,B). The pigs in the SVA CH/AH-02/2017 group showed moderate or severe necrosis, and the epithelial cells of necrotic foci exhibited swelling in the nasolabial skin and hoof epidermis. The histopathological lesions observed in the nasolabial skin and hoof epidermis were abundant neutrophil infiltrations, and a number of fragments of inflammatory cells existed in tissue (Figure 6C,D). 

### 3.6. Detection of SVA Viremia Levels and Viral Shedding

**Figure 7:**
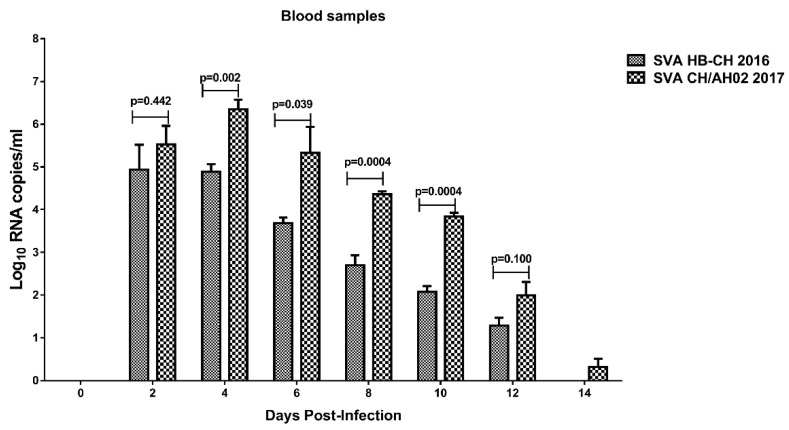
Comparison of viremia in the blood of pigs infected with the SVA HB-CH-2016 and SVA CH/AH-02/2017 strains. Viremia levels in the blood were determined by quantitative reverse transcription-polymerase chain reaction. The *p* value was calculated by the GraphPad Prism software. All data were expressed as mean ± SEM.

The total RNA was extracted from the blood, nasal swab, and fecal swab samples, and qRT-PCR was performed to detect SVA RNA, as previously described [10]. The qRT-PCR results showed that SVA RNA was detected in all blood samples from pigs infected with the SVA HB-CH-2016 and SVA CH/AH-02/2017 groups (Figure 7). This experiment confirmed that the experimental SVA strains could infect and replicate in pigs. 

**Figure 8:**
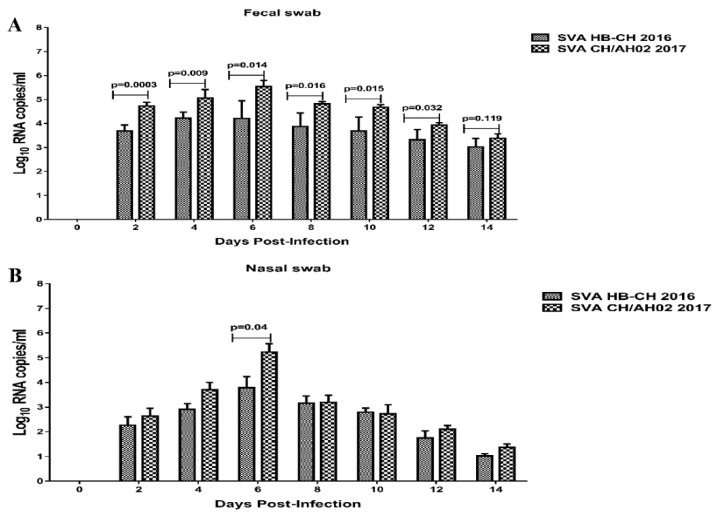
Viral shedding in pigs infected with the SVA HB-CH-2016 and SVA CH/AH-02/2017 strains. Viral shedding in the fecal swab (**A**) and nasal (**B**) samples of both the SVA HB-CH-2016 and SVA CH/AH-02/2017 groups was determined by RT-qPCR. The p value was calculated by GraphPad Prism software. All data were expressed as mean ± SEM.

Viremia was significantly higher in the SVA CH/AH-02/2017 group than in the SVA HB-CH-2016 group. A significant difference between the SVA HB-CH-2016 and SVA CH/AH-02/2017 groups was observed at 4, 6, 8, and 10 dpi (*p* < 0.05) (Figure 7). The viremia levels peaked at 4 dpi in both groups, with RNA loads of 10^4.885^ and 10^6.35^ copies/mL. Viral shedding was evaluated in nasal swab and fecal swab samples from infected pigs, both the SVA HB-CH-2016 and SVA CH/AH-02/2017 groups. The highest viral shedding on nasal and fecal swab samples of both the SVA HB-CH-2016 and SVA CH/AH-02/2017 groups was detected at 6 dpi (Figure 8). Following the challenge, the levels of viral shedding in nasal swab samples were similar between both groups. However, a significant difference was found in SVA RNA between the two groups at 6 dpi (*p* < 0.05) (Figure 8). An increased level of SVA RNA was detected in fecal swab samples from pigs in the SVA HB-CH-2016 and SVA CH/AH-02/2017 groups. A significant difference was found between the two groups.

### 3.7. Cross-Neutralization between SVA HB-CH-2016 and SVA CH/AH-02/2017

**Figure 9:**
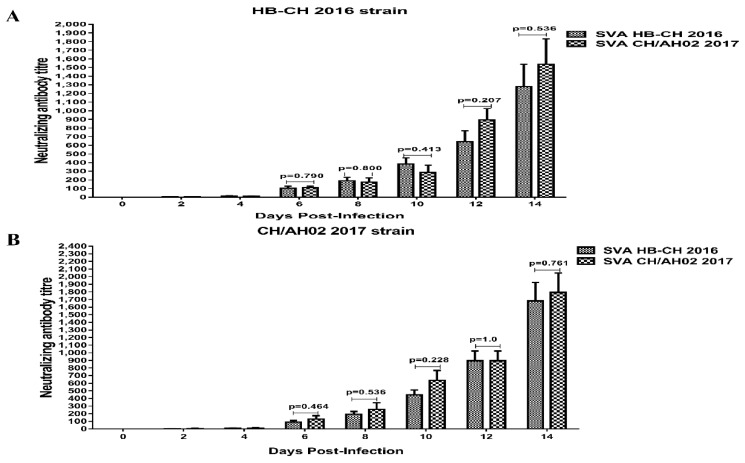
Comparison of the neutralizing antibody titers in serum samples from pigs infected with the SVA HB-CH-2016 and SVA CH/AH-02/2017 strains. The neutralizing antibody titers against the SVA HB-CH-2016 (**A**) and SVA CH/AH-02/2017 strains (**B**) were calculated and expressed as the highest dilution was inhibited completely. The *p* value was calculated by the GraphPad Prism software (GraphPad Software Inc., La Jolla, CA, USA). All data were expressed as mean ± SEM.

We analyzed all serum samples for SVA neutralizing antibodies to assess the serological responses. At 4 dpi, all infected pigs of both groups seroconverted (Figure 9). The neutralizing antibody level in both groups increased substantially following SVA challenge. The neutralizing antibody titer peaked at 14 dpi, and the mean neutralization antibody titer exceeded 1024. The mean neutralization titers induced by SVA CH/AH-02/2017 were higher against the homologous virus strain than against the heterologous virus strain. No significant difference was found in the antibody level between the SVA HB-CH-2016 and SVA CH/AH-02/2017 groups (*p* > 0.05) (Figure 9). Interestingly, SVA CH/AH-02/2017 induced high-level neutralizing antibody against the homologous and heterologous viral strains during early-stage infection. The abovementioned results indicated that both the SVA CH/AH-02/2017 and SVA HB-CH-2016 strains could infect pigs, and the different branches of SVA could induce broad cross-reactive neutralizing antibodies.

## 4. Discussion

In 2015, SVA was first reported by Wu Q et al. in China [15]. Thereafter, an increased number of cases of SVA have been reported in China, including Guangdong, Hubei, Guangxi, Fujian, Henan, Anhui, and Heilongjiang [6,16,17,18,19,20]. SVA can cause idiopathic vesicular disease in pigs. It is an emerging infectious disease that causes severe harm to the pig industry. At present, about 56 SVA strains with full genome sequences have been sequenced and reported in China. The phylogenetic tree showed different genotypes of SVA strains in China [20]. We further determined that all Chinese strains could be divided into two branches in a phylogenetic tree, namely, USA-like and Canadian-like strains. Since 2017, the USA-like SVA strains have become the dominant epidemic strains in China [6]. However, the pathogenicity of different strains of SVA in China has not been systematically studied. 

In this study, we compared the replication ability of different branches of SVA by using a porcine cell line and evaluated its pathogenicity in growing-finishing pigs. SVA HB-CH-2016 exhibited similar replication levels on SK-6 cells to the SVA CH/AH-02/2017 strain. The plaque formation assays and one-step growth experiment on SK-6 cells showed no distinct differences in plaque size and replication ability among different strains of SVA. We observed similar characteristic virions in both strains by electron microscopy. Multiple sequence alignment indicated that the different strains of SVA shared 94–99% identity at the nucleotide level in GenBank. 

Several recent studies have reported on the pathogenicity of SVA [5,7,21,22,23,24]. Theoretically, all SVA strains isolated from diseased pigs have pathogenic potentiality. However, under the infectious conditions of artificial simulations, some isolates are not pathogenic in pigs [15]. In the present study, the SVA HB-CH-2016 and SVA CH/AH-02/2017 strains were isolated from diseased adult pigs. Experimental results showed that both the SVA HB-CH-2016 and SVA CH/AH-02/2017 strains successfully infected growing-finishing pigs. However, substantial differences were found in the pathogenicity between the SVA HB-CH-2016 and SVA CH/AH-02/2017 strains. The pigs infected with the SVA CH/AH-02/2017 strain displayed a mild fever at 4 or 5 dpi and developed characteristic clinical signs compared with those infected with the SVA HB-CH-2016 strain. Throughout the experiment, the SVA HB-CH-2016 group did not present clinical signs and vesicular lesions. RT-PCR analysis showed that viremia persisted for 10 days in the blood in both groups. Both groups of pigs developed neutralizing antibody responses at 4 dpi, which increased with time upon inoculation with SVA. Interestingly, high levels of neutralizing antibodies were correlated with viral load reduction in the blood, and this finding was consistent with the results of previous studies [5,13,20,23]. These results indicated that neutralizing antibodies played an important role in clearing the virus of SVA. Similar levels of viral shedding were observed in the nasal secretions and feces of the two groups. Viral load was still detected in the feces of the two groups at 14 dpi. All these experiments supported that the SVA CH/AH-02/2017 strain is a highly pathogenic strain. The SVA HB-CH-2016 strain belongs to branches of Canadian-like strains, showing that the isolate had low pathogenicity to pigs. Similar results were observed in nine-week-old pigs (data not shown). 

Two different branches of SVA were selected to investigate pathogenicity in the present study. We found obvious differences in the pathogenicity and dynamics of infection among different genotypes of SVA strains in pigs. At present, USA-like SVA strains are the dominant pandemic strain in China [6]. The pathogenesis of SVA CH/AH-02/2017 was similar to that of the published results of USA strains. Our results confirmed that different pathogenicity of SVA strains exists in pig farms in China. The genetic diversity may explain the different biological properties. Therefore, the epidemic status of SVA should be constantly tested, and effective vaccines for controlling SVA infection in swine in China should be developed.

## Figures and Tables

**Table 1:** The differences in the amino acid between the two strains.

Protein	Positions (AA)	SVA HB-CH2016	SVACH/AH-02 2017
protein L	56	R	K
protein VP4	83	Q	H
protein VP2	426	Y	F
	427	K	T
protein VP3	494	P	S
	511	T	A
	575	T	A
protein VP1	738	T	A
	767	S	N
	845	A	7
	904	A	V
	941	V	I
protein 2C	1079	K	T
	1148	S	G
protein 3A	1401	N	T
	1428	Q	P
	1429	E	D
	1462	R	N
	1469	A	T
protein 3C	1589	V	L
	1647	E	D
	1649	T	S
	1685	L	M
protein 3D	1850	V	A
	1856	A	P
	2023	I	N
	2035	L	P
	2079	K	R
	2158	R	H
